# Unique North American isolates of severe metastatic hypervirulent *Klebsiella pneumoniae* strain infections with hepatic abscesses seen in young patients within Texas

**DOI:** 10.1371/journal.pone.0308305

**Published:** 2025-02-03

**Authors:** Junaid M. Alam, Eric N. Riha, Haris Ahmed, Hong M. Thai, Kevin Garnepudi, Ramesh B. Kesavan, Gnananandh Jayaraman, Anna Sangster, Dylan Curry, Heidi A. Butz, Lori Smith, Maureen Vowles, Kelly F. Oakeson, Erin L. Young, Siva T. Sarva

**Affiliations:** 1 Internal Medicine, HCA Houston Healthcare, Kingwood, TX, United States of America; 2 Infectious Disease, HCA Houston Healthcare, Kingwood, TX, United States of America; 3 Pulmonary and Critical Care Medicine, HCA Houston Healthcare Kingwood, Kingwood, TX, United States of America; 4 Pulmonary Critical Care and Sleep Specialists PA, Kingwood, TX, United States of America; 5 Utah Department of Health/ Utah Public Health Laboratory, Taylorsville, Utah, United States of America; Tianjin University, CHINA

## Abstract

**Rationale:**

Hypervirulent *Klebsiella pneumoniae* (hvKp) infections have principally been identified in Asia. Within a two-month period, two patients between the ages of 30 to 50 years old presented to a tertiary referral hospital in Texas with septic shock, hepatic abscess, and septic thrombophlebitis. Blood cultures were positive for *Klebsiella pneumoniae* (isolates 2020CK-00441 and 2021CK-00720 respectively). The first patient survived after a prolonged hospital course while the second patient expired.

**Objectives:**

Describe the clinical presentation of these two patients. Perform whole genome sequencing and bioinformatic analysis to evaluate potential outbreak of specific hvKp bacteria isolates.

**Methods:**

Whole genome sequencing was performed using both paired-end Illumina MiSeq and nanopore sequencing to obtain a Completed genome for both isolates.

**Main results:**

2020CK-00441 belonged to ST23 type while 2021CK-00720 was a ST65 type isolate. Kleborate analyses predicted with high confidence both isolates were hvKp. Phylogenetic analyses showed the two strains are not closely related to each other nor to any known hvKp isolates reported. Both isolates had yersiniabactin, colibactin, aerobactin and salmochelin producing loci which likely confer these isolates hvKp phenotype. 2020CK-00441 and 2021CK-00720 had a unique pK2044 like plasmid.

**Conclusions:**

HvKp strains capable of causing devastating metastatic septic infections have emerged in Texas. These isolates are unique compared to other hvKp strains globally. Country-wide surveillance and whole genome sequencing of these strains is essential to prevent a major public health emergency in the USA.

## Introduction

Hypervirulent *Klebsiella pneumoniae* (hvKp) was first recognized in Taiwan in 1986 [1]. Unlike the classical *Klebsiella pneumoniae* (cKp), hvKp affects healthy immunocompetent patients in the community setting rather than being nosocomial [2,3]. HvKp infection presents with fever, abdominal pain, and multiple hepatic abscesses. These abscesses often metastasize to multiple body sites leading to septic emboli, endophthalmitis, brain abscesses, and epidural abscesses. While most cases of hvKp are reported in Southeast Asia, it is an emerging pathogen in the Western countries [4–8]. Multi drug resistant and extensively drug resistant hvKp strains are emerging around the world [9–12].

Initially it was believed, hypermucoviscous phenotype identified by a “string test” was diagnostic of hvKp [13]. However, many hvKp isolates are not hypermucoviscous and limited cKp isolates are hypermucoviscous suggesting this test is unreliable for characterizing hvKp [14,15]. Furthermore, due to the presence of K1 and K2 capsule types in the hvKp isolates, capsule type alone can not be used to predict hvKp [16].

*Klebsiella pneumoniae* has been classified into many sequence types (ST types) using multi-locus sequence typing (MLST) and various clonal groups (CG groups) based on whole genome sequencing (WGS) [3]. Putative virulence factors, a) plasmid associated loci, iro (salmochelin biosynthesis), iuc (aerobactin synthesis), rmpA (regulator of mucoid phenotype); b) Integrative and Conjugative Element *Klebsiella pneumonia* (ICEKp) associated loci yersiniabactin and colibactin; are found to be more prevalent in hvKp isolates [3,15].

HvKp has the potential to become a major public health problem. Being conscious of this threat, isolates of the *Enterobacterales* order, carrying one or more genetic markers of hypervirulence, including peg-344, iroB, iucA, rmpA, and rmpA2, have been added to the list of AR/HAI alerts for public health laboratories within the Antibiotic Resistance Laboratory Network (ARLN). There is no consensus regarding the clinical and genetic characteristics that determine an isolate to be hvKp. Large scale studies in the United States have focused on limited genomic analyses of available isolate collections [17,18]. Within the U.S., whole genome sequences of hvKp clinical isolates have been described previously but do not appear to be isolated from patients suffering life threatening metastatic infections [19,20]. A clear enumeration of the clinical presentation, whole genome sequencing, and epidemiological analyses of the prevalent hvKp isolates in the United States is needed. To the best of our knowledge, for the first time in the U.S., we report two whole genome sequences of hvKp which caused severe metastatic life-threatening septic infections. Some of the results of these studies have been previously reported in the form of an abstract at American Thoracic Society International Conference 2021 [21].

## Methods

### Clinical cases

Consent was obtained from the patient and/or patient family to publish the clinical findings of the patients verbally and documented in the clinical chart. Written consents were not obtained due to the patients being extremely critical and in life threatening condition. The study and the consent procedures were approved by C.A.R.R.I.E. (Centralized Algorithms for Research Rules on IRB Exemption) of HCA Health Care and by “Pubclear’ the research review committee of HCA Houston Health Care, Kingwood.

### Isolate preparation and antibiotic sensitivity determination

Blood cultures were obtained using standard collection techniques and processed with Bactec FX system. Antibiotic sensitivities were obtained using both Microscan Walkaway system and Sensititre GNX2F panels (Thermo-Fisher Scientific). The modified carbapenem inactivation method (mCIM) was used to identify phenotypic carbapenemase production. Details of these methodologies are provided in Supplemental Methods A, B and C.

### Whole genome sequencing and bioinformatics

Bacterial isolates were grown on trypic soy agar with 5% sheep blood overnight. DNA was extracted using the Qiagen EZ1 DNA Tissue Kit (953034). WGS for 2020CK-00441 was done on both the Illumina MiSeq with Nextera DNA flex library preparation kits (20018705) [22], and Oxford Nanopore GridION with the Ligation Sequencing kit for the flongle adapter (FLO FLG001;SQK-LSK109) in accordance to manufacturer’s instructions. Fastq files are available from the SRA database as SRR13075498. A closed genome for 2020CK-00441 was obtained by utilizing a hybrid assembly of both long reads and short reads using Unicycler [23], and annotated with PGAP.

2021CK-00720 only underwent short-read Illumina sequencing. Fastq files are available from the SRA database as SRR13965555. Fastq reads were filtered for quality with Seqyclean a comprehensive preprocessing software pipeline [24]. Cleaned reads were *de novo* assembled with shovill (unpublished)(https://github.com/tseemann/shovill) into contigs. Bioinformatic hypervirulence prediction was performed via Kleborate, a tool to screen genome assemblies of *Klebsiella pneumoniae complex* (https://www.biorxiv.org/content/10.1101/2020.12.14.422303v2).

### Genome and plasmid comparisons

2020CK-00441 and 2021CK-00720 were compared to each other and the hypervirulent Klebsiella genomes downloaded from BioProjects PRJNA506506, PRJNA509089, PRJNA509091, PRJEB38367, PRJNA349219, PRJNA391211, PRJNA638288 and PRJEB34922. ST23 and ST65 typing was ascertained from kleborate, contig and genome fasta files were annotated with Prokka and aligned with Roary, a pan genome pipeline, into a core genome alignment [25,26]. Snp-dists software was used to count the number of SNPs between the two described isolates (unpublished)(https://github.com/tseemann/snp-dists). Phylogenetic trees were generated with iqtree and visualized with ggtree [25–28]. The blast web portal (https://blast.ncbi.nlm.nih.gov/Blast.cgi) was used to compare p2020CK-00441_1, p2021CK-00720_1, and pK2044 in March 2022 [29]. Mash distances to the sequences in PLSDB [30,31], a curated plasmid database containing 34513 unique sequences, were performed via the portal (https://ccb-microbe.cs.uni-saarland.de/plsdb/, code version v0.4.1-386-gd7e4b70b05 data version 2021_06_23_v2 (code v0.2, DBs 2021_06_23)). Mash distances reduce a target and subject genome into a representative sketch to estimate global mutation distances (https://genomebiology.biomedcentral.com/articles/10.1186/s13059-016-0997-x) and are commonly used to assess similarity of two sequences. Histograms and bar plots were created with ggplot2, arranged with ggpubr, and colored with ggsci in R.

## Results

### Clinical presentation

#### Case #1

**Fig 1:**
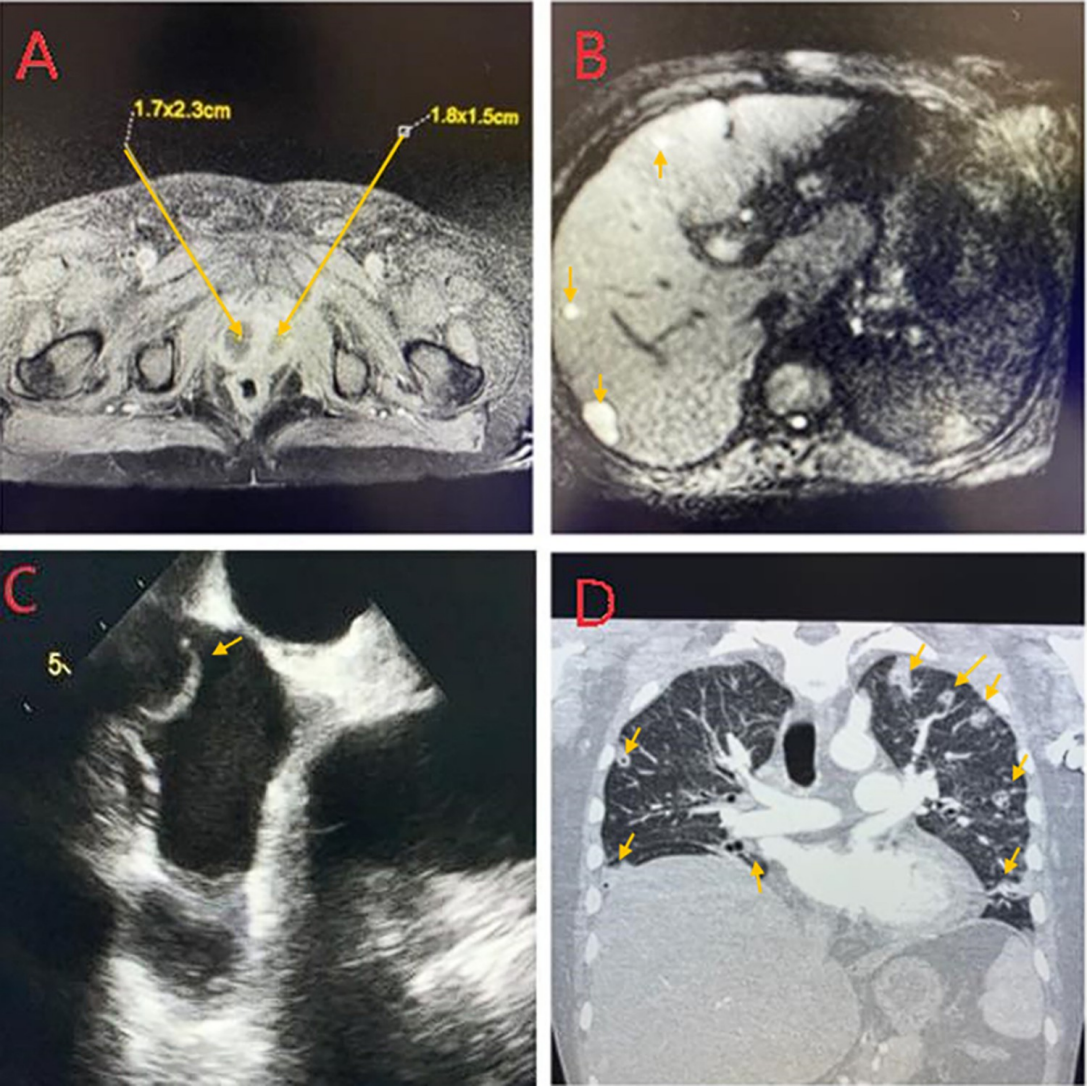
Metastatic septic infection by hypervirulent *Klebsiella pneumoniae* (2020CK-00441). Panel A: MRI Pelvis showing bilateral prostatic abscesses measuring 1.7x2.3cm on right and 1.8x1.5cm on left. Panel B: MRI Abdomen series showing multiple small hepatic abscesses throughout the liver. Panel C: Trans-esophageal echocardiogram showing a clot traversing the right atrium from the inferior vena cava. Panel D: CT imaging of chest showing multiple septic emboli throughout bilateral lung fields. Pathology identified by yellow arrow in each panel.

A patient in the age group of 30 to 50 years-old with a past medical history of diabetes mellitus presented from home via Emergency Medical Services unresponsive with collateral history of abdominal pain and dysuria for 2 weeks. On arrival, the patient was hypoxic, tachycardic, and hypotensive. Physical examination revealed diffusely tender abdomen and coarse breath sounds. Laboratory values were significant for elevated lactate (11.8 mmol/L) and thrombocytopenia (112 x 10^3^ /ul) (S1 Table in S1 Data). CT imaging of the chest and abdomen revealed extensive pulmonary septic emboli, prostate abscesses, right pyelonephritis, multiple hepatic abscesses, and thrombus within the inferior vena cava (Fig 1). The patient was subsequently admitted to the intensive care unit and managed with appropriate fluid resuscitation, vasopressors, mechanical ventilator support, meropenem, and heparin drip.

Blood cultures grew *Klebsiella pneumoniae* (2020CK-00441). Transesophageal echocardiogram showed a clot in the inferior vena cava projecting into the right atrium and no evidence of vegetation or paravalvular abscess (Fig 1). The patient was extubated after three days and weaned from vasopressor support within one week. MRI of the abdomen and pelvis showed multiple hepatic abscesses and was negative for gall stones, psoas abscess, or pelvic osteomyelitis (Fig 1). MRI of the brain, cervical, thoracic and lumbar spine were negative for abscesses and stroke. The patient was discharged in stable condition after 16 days.

#### Case #2

A patient in the age group of 30 to 50 years old with diabetes mellitus and hypertension presented with current right abdominal and back pain and a syncopal episode 10 days prior. This patient had associated headache, poor appetite, dizziness, and back pain prompting the visit to the emergency room. On admission, the patient was afebrile, tachycardic (103 /min), blood pressure of 116/76 mm Hg, soft abdomen, supple neck, and clear lung sounds bilaterally. Initial laboratory values were significant for elevated neutrophil band cells (42%), thrombocytopenia (50 x 10^3^ /ul), arterial blood gas with normal pH, and mild lactate elevation (2.3 mmol/L) (S1 Table in S1 Data). CT head without contrast was negative for acute abnormality. CT imaging of the chest and abdomen showed septic emboli in the lung, enlarged liver with high suspicion for a liver abscess, and hepatic vein thrombosis. The patient was admitted to the Intermediate Medical Unit and started on Piperacillin/Tazobactam, basal insulin, and maintenance fluids. In the presence of severe thrombocytopenia, full dose anti-coagulation was not initiated.

Six hours from presentation, the patient became hypotensive and encephalopathic. Focal weakness or seizure-like activity were not observed. Laboratory values were significant for worsening lactic acidosis (8.8 mmol/L) and thrombocytopenia (30 x 10^3^ /ul). The Critical Care team was consulted, and the patient was transferred to the Intensive Care Unit. Appropriate fluid resuscitation, insulin drip, mechanical ventilation and vasopressors were started. Unfortunately, 20 hours from presentation the patient experienced cardiac arrest and subsequently expired after more than an hour of cardiopulmonary resuscitation. Two days later, the blood cultures were positive for *Klebsiella pneumoniae* (2021CK-00720) while urine cultures remained negative.

### Clinical laboratory

Antibiotic sensitivity testing was performed on both isolates, described in the methods. Multi-drug resistance, carbapenem resistance, and carbapenemases were not observed in either isolate by modified carbapenem inactivation method (mCIM) testing. The detailed antibiotic sensitivities and minimal inhibitory concentration profiles using routine laboratory testing and alternative Sensititre GNX2F panel testing for both isolates are provided in Supplemental Methods B, C and S2 and S3 Tables in S1 Data.

### Basic science

#### Whole genome sequencing

Hybrid assembly of 2020CK-00441 resulted in one closed chromosome sequence, two closed plasmid sequences, and no known incomplete plasmid sequences. PGAP identified 5,338 genes and 130 pseudogenes in the assembly. Details of the annotation are provided in S4 Table in S1 Data. Loci and genes associated with hypervirulence were identified on the chromosomal sequence resembling the ICEKp10 mobile element and p2020CK-00441_1.

Hybrid assembly of 2021CK-00720 resulted in one closed chromosome sequence and one closed plasmid sequences, and no known incomplete plasmid sequences. PGAP identified 5,455 genes and 123 pseudogenes in the assembly. Details of the annotation are provided in S4 Table in S1 Data. Loci and genes associated with hypervirulence were identified on the chromosomal sequence resembling the ICEKp10 mobile element and p2021CK-00720_1.

Both strains were given a score of five for virulence by Kleborate analysis which shows high probability of the isolates to be hvKp strains (Table 1). Neither isolate had genomic evidence of multi-drug resistance or carbapenam resistance (S2 Table in S1 Data). 2020CK-00441 had ybt1-clb2 profile while 2021CK-00720 had ybt17-clb3 profile. Both isolates had virulence genes encoding aerobactin (iuc1), salmochelin (iro1), and rmpADC loci which are frequently present in pK2044 like plasmids in hvKp strains. The presence of rmpADC loci in both isolates suggests both are likely to have a hypermucoviscous phenotype.

**Table 1:** Summary of kleborate analysis.

Isolate	2020CK-00441	2021CK-00720
**Species**	Klebsiella pneumoniae	Klebsiella pneumoniae
**ST**	ST23	ST65
**Virulence Score**	5	5
**Resistance Score**	0	0
**Number of Drug Resistance Genes**	0	0
**Yersiniabactin**	ybt 1; ICEKp10	ybt 17; ICEKp10
**Colibactin**	clb 2	clb 3
**Aerobactin**	iuc 1	iuc 1
**Salmochelin**	iro 1	iro 1
**rmpADC**	rmp 1; KpVP-1	rmp 1; KpVP-1
**rmpA2**	rmpA2_6–60%	rmpA2_5–54%
**wzi**	wzi1	wzi72
**K_locus**	KL1	KL2
**O_type**	O1	O1

Summary of kleborate analysis showing that both the isolates are highly likely to be hvKp (Virulence score = 5) and unlikely to be multi-drug resistant (Resistance Score = 0).

#### Comparison of p2020CK-00441_1 and pK2044, p2020CK-00441_2, and p2021CK-00720

**Fig 2:**
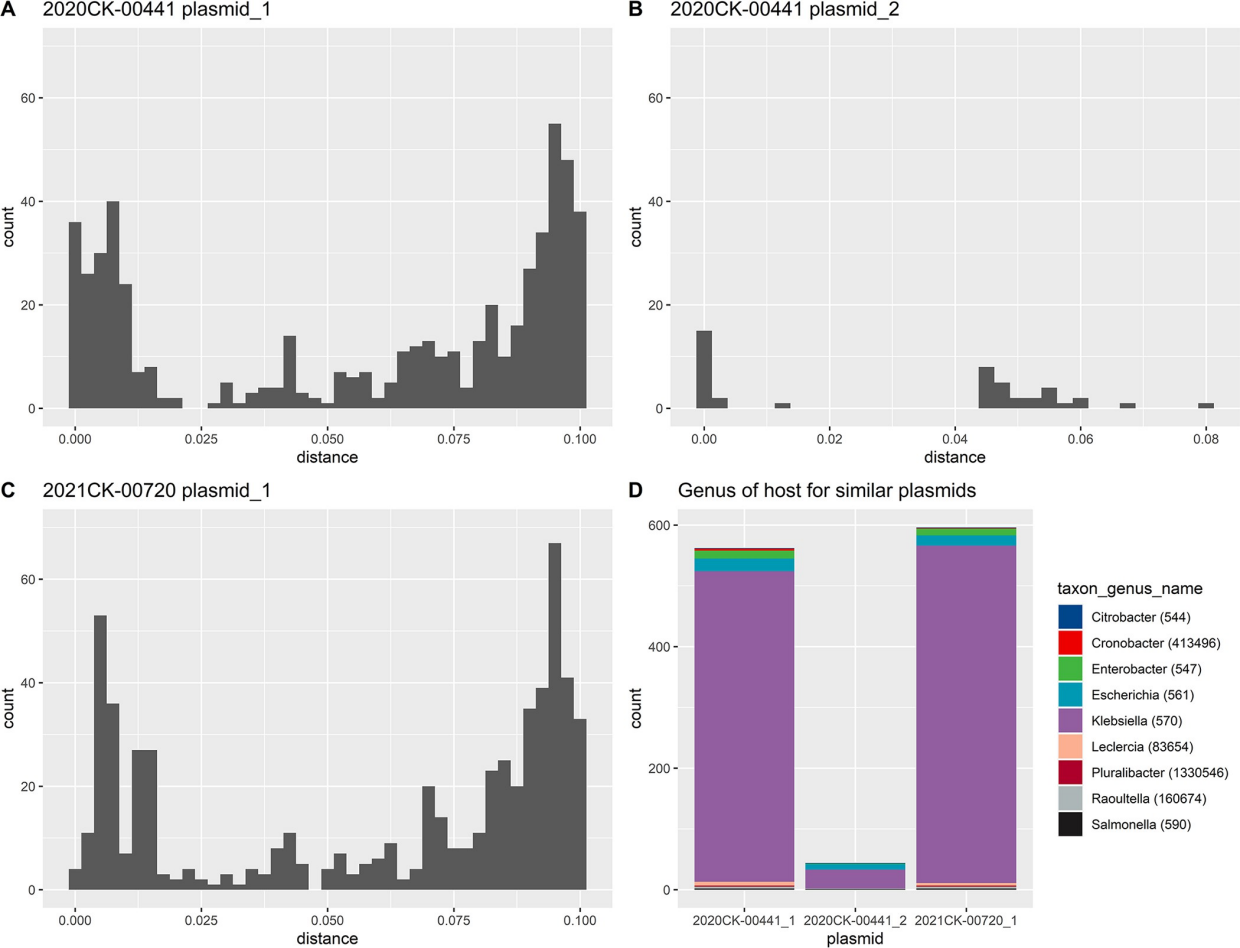
Histogram of mash distances identified from A) p2020CK-00441_1, B) p2020CK-00441_2, and C) 2021CK-00720_1 when compared to PLSDB with p-value = 0 and mash distance < = 0.1 thresholds. D) Stacked bar graph of genus associated with the PLSDB plasmids identified in A-C.

p2020CK-00441_1 is a non-self-transmissible IncFIBK type virulence plasmid. When compared to previously described pK2044 plasmid, it is nearly identical (99.98% identity and 98% coverage via the blast web portal). p2021CK-00720_2 was also found to be similar to p2020CK-00441_1 (99.5% identity and 95.0% coverage) and pK2044 (99.8% identity and 94.0% coverage) 2It was worth investigating the similarity between the identified plasmids and those published previously as curated by the PLSDB (30, 31).2020CK-00441_1 had a distance of less than 0.1 to 562 other plasmids (p-value = 0), and 90.9% were mostly identified from sequencing Klebsiella. 2021CK-00720_1 was similar to 596 plasmids, 93.1% of which were from Klebsiella species (Fig 2 and S4 Table in S1 Data).

The second smaller plasmid of 2020CK-00441p2020CK-00441_2 is nearly identical to previously described ColRNAI type *Klebsiella* plasmids, pUUH239.1 and pBK13048_7 (99% identity and 100% coverage as determined via the blast web portal). These plasmids encode for plasmid mobilization proteins and phage resistance proteins and their role is not yet defined in HvKp phenotype. 020CK-00441_2 was only similar to 40 plasmids under the same constraints as 2020CK-00441_1 (Fig 2B and 2D), of which only 70.5% plasmids were found from sequencing Klebsiella and 20.5% from Escherichia species.

#### ICE regions of 2020CK-00441

**Fig 3:**
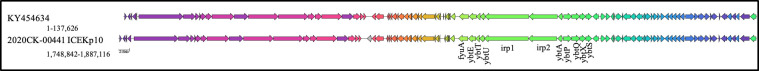
Synteny alignment of KY454634,of the ICEKp10 region of 2020CK-00441, the ICEKp10 region of 2021CK-00720 extended to match the length of the other two sequences. Hypervirulent genes are annotated with text. Synteny groups share colors. Shading between sequences indicates >90% identity.

Integrative Conjugative elements (ICE) are self-transmissible mobile genetic elements that are key mediators for gene flow for bacteria. 2020CK-00441 contains the ICEKp10 mobile element in its chromosome, which is typical of ST23 type Klebsiella [32,33]. When compared with KY454634, the arrangement of genes is almost an exact match, with the exception of a hypothetical protein with similarity to IS5 family transposase IS903 (Fig 3). It is unclear if this gene insertion impacts hypervirulence. 2021CK-00720 is also expected to contain the ICEKp10 mobile element by kleborate analysis, but this could not be confirmed due to the limitations of short-read Illumina sequencing.

Homology searches revealed an additional potential ICE element in 2020CK-0441 which is similar to *E*.*coli* ICEEc2. This region encodes for hypothetical genes and Type IV secretion system. None of these genes have been implicated in contributing to hvKp phenotype thus far.

#### Comparison of 2020CK-00441 and 2021CK-00720

Although no known hvKp cases were observed in the past decade at HCA Houston Healthcare, both 2020CK-00441 and 2021CK-00720 presented a few weeks apart. Although 2020CK-00441 and 2021CK-00720 MLST were identified as MLST subtypes ST23 and ST65 respectively, the identification of both Klebsiella in such a narrow time window prompted further investigation. 2020CK-00441 and 2021CK-0072 share 4,577 genes (out of 5,646 genes identified via prokka) with a combined length of 3922994 bases, but there were 29,575 SNPs (0.75%) in these genes when comparing the sequence of one isolate to the other. This suggested that the isolates a) were not closely related, b) were unlikely to besimilar in origin, and c) may be from more than one strain of hvKp present in the community around the hospital.

#### Phylogenetic tree analysis

**Fig 4:**
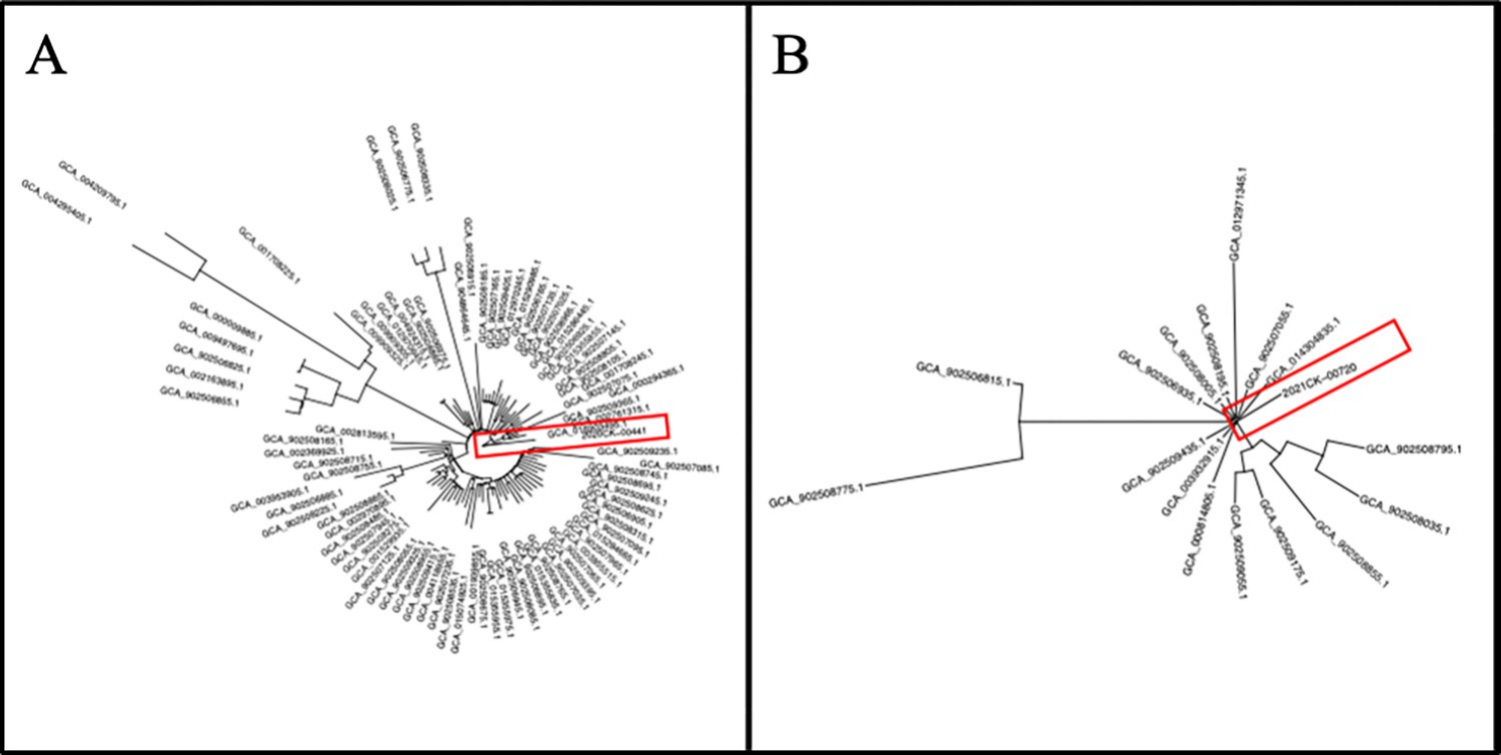
Phylogenetic trees of A) 2020CK-00441 (highlighted with red box) with available ST23 genomes and B) 2021CK-00720 (highlighted with red box) with available ST65 genomes.

To determine if the two observed cases were similar to other hvKp studies reported from other continents, a phylogenetic tree was created with the ST types of both of our isolates with previously reported hvKp Bioprojects (Fig 4). Surprisingly, the genomic sequences of both of the strains were not closely related to any of the other hvKp sequence submitted to NCBI thus far. This suggests that the two US isolates are distinct from the isolates in other continents and have the potential to have varying clinical and microbiological profiles.

### Epidemiology

In addition to a review of the hospital chart for social history, phone surveys with the patient and/or family was performed before the submission of this report to identify occupational or travel related exposures. One of the patients did not have any known sick contacts, occupational exposure, and or any international travel while the other patient had significant remote history of international travel.

## Discussion

In this report we were able to showcase two infections that were caused by vastly different hvKp bacterial strains in two patients from significantly different demographic backgrounds. Definitive conclusions regarding optimal clinical management cannot be made with such a small sample size. A detailed review of the two cases was performed by the physicians of our team. The general principles that we would recommend to clinicians in managing these patients include, a) early recognition of these infections, b) prompt admission to critical care unit, c) initial broad spectrum antibiotics, d) aggressive imaging to identify foci of infection, and e) therapeutic anti-coagulation.

Early recognition of these infections is vital. It would be beneficial for physicians in the United States to include hvKp metastatic septic infections in the differential diagnosis of all patients with septic emboli, especially in young, poorly controlled diabetics. Patients with encephalopathy, thrombocytopenia, and high number of immature neutrophils (band cells) may have poor prognosis and require intensive care admission. CT-imaging of the chest, abdomen, and pelvis may help identify large abscesses and disease extent. MRI of the abdomen and pelvis may be needed to delineate the abscesses in the abdomen and identify potential osteomyelitis. Dilated eye exam, lumbar puncture, and MRI of the brain and spine may be required for identifying neurologically septic infections. Both the patients showed thrombophlebitis and thrombosis of major veins in the abdomen, a likely source of metastatic septic emboli to the lung. Therapeutic anti-coagulation may be beneficial.

The two patients described in this report do not share any common epidemiological risk factors or known connection. With no known exposure or travel history, one of the patients appears to have acquired the disease in the community. The other patient’s infection cannot be exclusively attributed to community spread as the patient had a significant international travel history.

Chou et al. screened 64 *Klebsiella pneumoniae* isolates for putative hvKp genes rmpA and magA in two major hospitals of Houston, Texas (about 25 miles south of our hospital) [18]; this is the only publication describing hvKp in Texas that we could identify from literature search. Only one of the four isolates was associated with liver abscesses while the others did not cause clinically significant infections. The cases did not have a metastatic septic infection, and the isolates did not undergo WGS. Whether these isolates indicate the presence of strains causing milder disease which have evolved into more severe forms or were newly introduced to the community is unknown at this time.

In this report we show that both 2020CK-0041 and 2021CK-00720 have genomic content necessary to make additional siderophores like yersiniabactin, aerobactin, and salmochelin. Highly virulent strains in other bacterial species have been shown to have higher expression of siderophore producing genes when compared to less virulent strains of the same species [34,35]. These factors are postulated to give these strains an advantage to obtain iron and counteract the innate defense mechanisms of the human immune system. Whether these siderophores are synthesized and if these siderophores play a role in the causation of the metastatic septic disease need future studies.

From a public health perspective, surveillance of these organisms and hypervirulence factors with WGS is needed to adequately inform response and infection prevention. The addition of *Klebsiella* hypervirulence genetic markers on the list of public health alerts reportable by laboratories within the ARLN, is a timely and welcome inclusion.

As one of the major hospitals serving the communities who live close to and work in Houston’s Bush Intercontinental Airport, it is possible that we may be seeing index cases of locally adapted hvKp strains capable of causing devastating metastatic septic infections. By transfer of relatively short genomic sequences, there is a potential for emergence of many locally adapted hvKp strains. To understand the rate of emergence and spread of problematic strains, a geographically based detailed catalog of the genomic sequences of both cKP and hvKp is essential. A seamless “patient to laboratory to bench to community” translational approach with the close collaboration of physicians, clinical microbiologists, experts in basic science, epidemiologists, and the health department is paramount in the prevention of this emerging threat.

## Supporting information

S1 Data (DOCX)

S1 File (DOCX)

## References

[1] Liu YC, Cheng DL, Lin CL. Klebsiella pneumoniae liver abscess associated with septic endophthalmitis. Archives of internal medicine. 1986;146(10):1913–6. Epub 1986/10/01. 3532983

[2] Russo TA, Marr CM. Hypervirulent Klebsiella pneumoniae. Clinical microbiology reviews. 2019;32(3). Epub 2019/05/17. 10.1128/CMR.00001-19 PMC6589860 31092506

[3] Choby JE, Howard-Anderson J, Weiss DS. Hypervirulent Klebsiella pneumoniae—clinical and molecular perspectives. Journal of internal medicine. 2020;287(3):283–300. Epub 2019/11/05. doi: 10.1111/joim.13007 31677303 PMC7057273

[4] Lederman ER, Crum NF. Pyogenic liver abscess with a focus on Klebsiella pneumoniae as a primary pathogen: an emerging disease with unique clinical characteristics. The American journal of gastroenterology. 2005;100(2):322–31. Epub 2005/01/26. doi: 10.1111/j.1572-0241.2005.40310.x 15667489

[5] Pomakova DK, Hsiao CB, Beanan JM, Olson R, MacDonald U, Keynan Y, et al. Clinical and phenotypic differences between classic and hypervirulent Klebsiella pneumonia: an emerging and under-recognized pathogenic variant. European journal of clinical microbiology & infectious diseases: official publication of the European Society of Clinical Microbiology. 2012;31(6):981–9. Epub 2011/09/16. 10.1007/s10096-011-1396-6 21918907

[6] Decre D, Verdet C, Emirian A, Le Gourrierec T, Petit JC, Offenstadt G, et al. Emerging severe and fatal infections due to Klebsiella pneumoniae in two university hospitals in France. Journal of clinical microbiology. 2011;49(8):3012–4. Epub 2011/06/17. doi: 10.1128/JCM.00676-11 21677064 PMC3147753

[7] Sobirk SK, Struve C, Jacobsson SG. Primary Klebsiella pneumoniae Liver Abscess with Metastatic Spread to Lung and Eye, a North-European Case Report of an Emerging Syndrome. The open microbiology journal. 2010;4:5–7. Epub 2010/05/08. doi: 10.2174/1874285801004010005 20448814 PMC2864426

[8] Moore R O’Shea D, Geoghegan T, Mallon PW, Sheehan G. Community-acquired Klebsiella pneumoniae liver abscess: an emerging infection in Ireland and Europe. Infection. 2013;41(3):681–6. Epub 2013/02/06. 23381876 10.1007/s15010-013-0408-0

[9] Wei DD, Wan LG, Deng Q, Liu Y. Emergence of KPC-producing Klebsiella pneumoniae hypervirulent clone of capsular serotype K1 that belongs to sequence type 11 in Mainland China. Diagnostic microbiology and infectious disease. 2016;85(2):192–4. Epub 2016/04/07. doi: 10.1016/j.diagmicrobio.2015.03.012 27049969

[10] Feng Y, Lu Y, Yao Z, Zong Z. Carbapenem-Resistant Hypervirulent Klebsiella pneumoniae of Sequence Type 36. Antimicrobial agents and chemotherapy. 2018;62(7). Epub 2018/05/02. doi: 10.1128/AAC.02644-17 29712659 PMC6021629

[11] Zhang R, Lin D, Chan EW, Gu D, Chen GX, Chen S. Emergence of Carbapenem-Resistant Serotype K1 Hypervirulent Klebsiella pneumoniae Strains in China. Antimicrobial agents and chemotherapy. 2016;60(1):709–11. Epub 2015/11/18. doi: 10.1128/AAC.02173-15 26574010 PMC4704206

[12] Gu D, Dong N, Zheng Z, Lin D, Huang M, Wang L, et al. A fatal outbreak of ST11 carbapenem-resistant hypervirulent Klebsiella pneumoniae in a Chinese hospital: a molecular epidemiological study. The Lancet Infectious diseases. 2018;18(1):37–46. Epub 2017/09/03. doi: 10.1016/S1473-3099(17)30489-9 28864030

[13] Fang CT, Chuang YP, Shun CT, Chang SC, Wang JT. A novel virulence gene in Klebsiella pneumoniae strains causing primary liver abscess and septic metastatic complications. The Journal of experimental medicine. 2004;199(5):697–705. Epub 2004/03/03. doi: 10.1084/jem.20030857 14993253 PMC2213305

[14] Catalan-Najera JC, Garza-Ramos U, Barrios-Camacho H. Hypervirulence and hypermucoviscosity: Two different but complementary Klebsiella spp. phenotypes? Virulence. 2017;8(7):1111–23. Epub 2017/04/14. doi: 10.1080/21505594.2017.1317412 28402698 PMC5711391

[15] Russo TA, Olson R, Fang CT, Stoesser N, Miller M, MacDonald U, et al. Identification of Biomarkers for Differentiation of Hypervirulent Klebsiella pneumoniae from Classical K. pneumoniae. Journal of clinical microbiology. 2018;56(9). Epub 2018/06/22. 10.1128/JCM.00776-18 PMC6113484 29925642

[16] Holt KE, Wertheim H, Zadoks RN, Baker S, Whitehouse CA, Dance D, et al. Genomic analysis of diversity, population structure, virulence, and antimicrobial resistance in Klebsiella pneumoniae, an urgent threat to public health. Proceedings of the National Academy of Sciences of the United States of America. 2015;112(27):E3574–81. Epub 2015/06/24. doi: 10.1073/pnas.1501049112 26100894 PMC4500264

[17] Parrott AM, Shi J, Aaron J, Green DA, Whittier S, Wu F. Detection of multiple hypervirulent Klebsiella pneumoniae strains in a New York City hospital through screening of virulence genes. Clinical microbiology and infection: the official publication of the European Society of Clinical Microbiology and Infectious Diseases. 2020. Epub 2020/05/29. 10.1016/j.cmi.2020.05.012 32461145

[18] Chou A, Nuila RE, Franco LM, Stager CE, Atmar RL, Zechiedrich L. Prevalence of hypervirulent Klebsiella pneumoniae-associated genes rmpA and magA in two tertiary hospitals in Houston, TX, USA. Journal of medical microbiology. 2016;65(9):1047–8. Epub 2016/07/10. doi: 10.1099/jmm.0.000309 27392968 PMC5068137

[19] Kochan TJ, Ozer EA, Pincus NB, Fitzpatrick MA, Hauser AR. Complete Genome Sequence of Klebsiella pneumoniae Strain TK421, a Conjugative Hypervirulent Isolate. Microbiology resource announcements. 2020;9(3). Epub 2020/01/18. doi: 10.1128/MRA.01408-19 31948967 PMC6965585

[20] Prokesch BC, TeKippe M, Kim J, Raj P, TeKippe EM, Greenberg DE. Primary osteomyelitis caused by hypervirulent Klebsiella pneumoniae. The Lancet Infectious diseases. 2016;16(9):e190–e5. Epub 2016/07/13. doi: 10.1016/S1473-3099(16)30021-4 27402393

[21] Alam JM, Ahmed H, Thai H, Garnepudi K, Kesavan RB, Jayaraman G, et al. Severe Metastatic Klebsiella pneumoniae infection caused by hypervirulent strain ST23 in a 38 year old with hepatic abscess, septic thrombophlebitis and septic embolism. 2021.

[22] Ravi RK, Walton K, Khosroheidari M. MiSeq: A Next Generation Sequencing Platform for Genomic Analysis. Methods in molecular biology. 2018;1706:223–32. Epub 2018/02/10. doi: 10.1007/978-1-4939-7471-9_12 29423801

[23] Wick RR, Judd LM, Gorrie CL, Holt KE. Unicycler: Resolving bacterial genome assemblies from short and long sequencing reads. PLoS computational biology. 2017;13(6):e1005595. Epub 2017/06/09. doi: 10.1371/journal.pcbi.1005595 28594827 PMC5481147

[24] Zhbannikov IY, Hunter SS, Foster JA, Settles ML. SeqyClean: A Pipeline for High-throughput Sequence Data Preprocessing. Proceedings of the 8th ACM International Conference on Bioinformatics, Computational Biology,and Health Informatics; Boston, Massachusetts, USA: Association for Computing Machinery; 2017. p. 407–16.

[25] Seemann T. Prokka: rapid prokaryotic genome annotation. Bioinformatics. 2014;30(14):2068–9. Epub 2014/03/20. doi: 10.1093/bioinformatics/btu153 24642063

[26] Page AJ, Cummins CA, Hunt M, Wong VK, Reuter S, Holden MT, et al. Roary: rapid large-scale prokaryote pan genome analysis. Bioinformatics. 2015;31(22):3691–3. Epub 2015/07/23. doi: 10.1093/bioinformatics/btv421 26198102 PMC4817141

[27] Kalyaanamoorthy S, Minh BQ, Wong TKF, von Haeseler A, Jermiin LS. ModelFinder: fast model selection for accurate phylogenetic estimates. Nature methods. 2017;14(6):587–9. Epub 2017/05/10. doi: 10.1038/nmeth.4285 28481363 PMC5453245

[28] Yu G, Lam TT, Zhu H, Guan Y. Two Methods for Mapping and Visualizing Associated Data on Phylogeny Using Ggtree. Molecular biology and evolution. 2018;35(12):3041–3. Epub 2018/10/24. doi: 10.1093/molbev/msy194 30351396 PMC6278858

[29] Gilchrist CLM, Chooi YH. Clinker & clustermap.js: Automatic generation of gene cluster comparison figures. Bioinformatics. 2021. Epub 2021/01/19. 10.1093/bioinformatics/btab007 33459763

[30] Georges P Schmartz Anna Hartung, Hirsch Pascal, Kern Fabian, Fehlmann Tobias, Müller Rolf et al; PLSDB: advancing a comprehensive database of bacterial plasmids, Nucleic Acids Res., 2021 Nov 25, doi: 10.1093/nar/gkab1111 34850116 PMC8728149

[31] Galata Valentina, Fehlmann Tobias, Backes Christina, Andreas Keller; PLSDB: a resource of complete bacterial plasmids, Nucleic Acids Res., 2018 Oct 31, doi: 10.1093/nar/gky1050 30380090 PMC6323999

[32] Lam MMC, Wyres KL, Duchene S, Wick RR, Judd LM, Gan YH, et al. Population genomics of hypervirulent Klebsiella pneumoniae clonal-group 23 reveals early emergence and rapid global dissemination. Nature communications. 2018;9(1):2703. Epub 2018/07/15. doi: 10.1038/s41467-018-05114-7 30006589 PMC6045662

[33] Struve C, Roe CC, Stegger M, Stahlhut SG, Hansen DS, Engelthaler DM, et al. Mapping the Evolution of Hypervirulent Klebsiella pneumoniae. mBio. 2015;6(4):e00630. Epub 2015/07/23 doi: 10.1128/mBio.00630-15 26199326 PMC4513082

[34] Searle LJ, Meric G, Porcelli I, Sheppard SK, Lucchini S. Variation in siderophore biosynthetic gene distribution and production across environmental and faecal populations of Escherichia coli. PloS one. 2015;10(3):e0117906. Epub 2015/03/11. doi: 10.1371/journal.pone.0117906 25756870 PMC4355413

[35] Sarva ST, Waldo RH, Belland RJ, Klose KE. Comparative Transcriptional Analyses of Francisella tularensis and Francisella novicida. PloS one. 2016;11(8):e0158631. Epub 2016/08/19. doi: 10.1371/journal.pone.0158631 27537327 PMC4990168

