# Supplementary material for: Unique North American isolates of severe metastatic hypervirulent Klebsiella pneumoniae strain infections with hepatic abscesses seen in young patients within Texas
Source: PLoS One. 2025 Feb 3;20(2):e0308305. doi: 10.1371/journal.pone.0308305 (PMC11790080; doi:10.1371/journal.pone.0308305)
Supplement: S1 File — (DOCX) [file pone.0308305.s002.docx]

Two isolates of hypervirulent Klebsiella pneumoniae were sent for genome sequencing at the University of Utah. Below you will find fastq files from both of the cases analyzed in this manuscript. All samples are publicly available and have been submitted to the National Library of Medicine by the Utah Public Health Laboratory Infectious Disease submission group. We attest to their completeness and originality.

Isolate from Case 1: 2020CK-00441

SRR13075498

<https://www.ncbi.nlm.nih.gov/sra/?term=SRR13075498>

Isolate from Case 2: 2021CK-00720

SRR13965555

<https://www.ncbi.nlm.nih.gov/sra/?term=SRR13965555>
